# Hemi Orolingual Angioedema after tPA Administration for Acute Ischemic Stroke

**DOI:** 10.5811/westjem.2014.12.24210

**Published:** 2015-01-12

**Authors:** Bryan Madden, Ralphe B. Chebl

**Affiliations:** Henry Ford Hospital, Department of Emergency Medicine, Detroit, Michigan; American University of Beirut, Department of Emergency Medicine, Beirut, Lebanon

## INTRODUCTION

As studies continue to demonstrate the efficacy of intravenous tissue plasminogen activator (tPA) in acute ischemic stroke, the exclusion criteria continue to narrow, and the time-window continues to increase.^1^–^3^ The most dreaded adverse effect of tPA, hemorrhagic conversion of an ischemic stroke, is well known and well published.^3^ However, as an increasing number of patients are receiving tPA worldwide another unusual and potentially life-threatening adverse effect of tPA is becoming more common, angioedema.

### Case Report

A 50-year-old man with a history of hypertension, hyperlipidemia, crack-cocaine abuse, and two prior ischemic strokes (2004, 2011) characterized by right-sided weakness with no residual deficits presented to a large urban hospital with left-sided arm weakness, left-sided leg weakness, and mild slurring of speech. Patient had received tPA when he presented with his stroke symptoms in 2011, without any adverse events. He was last seen normal four hours prior to arrival. On presentation he had a National Institute of Health Stroke Scale (NIHSS) of 5, left-sided facial droop, drift and ataxia in both his left arm and left leg. His blood pressure was 175/103mmHg, a pulse of 94beats/minute, a temperature of 37.2°C and a right atrium oxygen saturation of 98%. A computed tomography of the head was negative for any acute hemorrhage. Patient was supposed to be on an angiotensin- converting enzyme (ACE) inhibitor but had been non compliant with his medications for the last three months.

**Figure 1:**
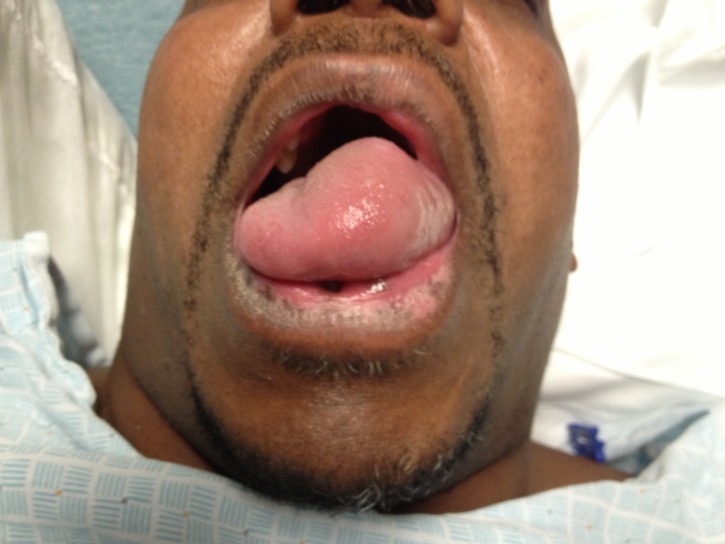
Hemi orolingual swelling after tissue plasminogen activator infusion.

**Figure 2:**
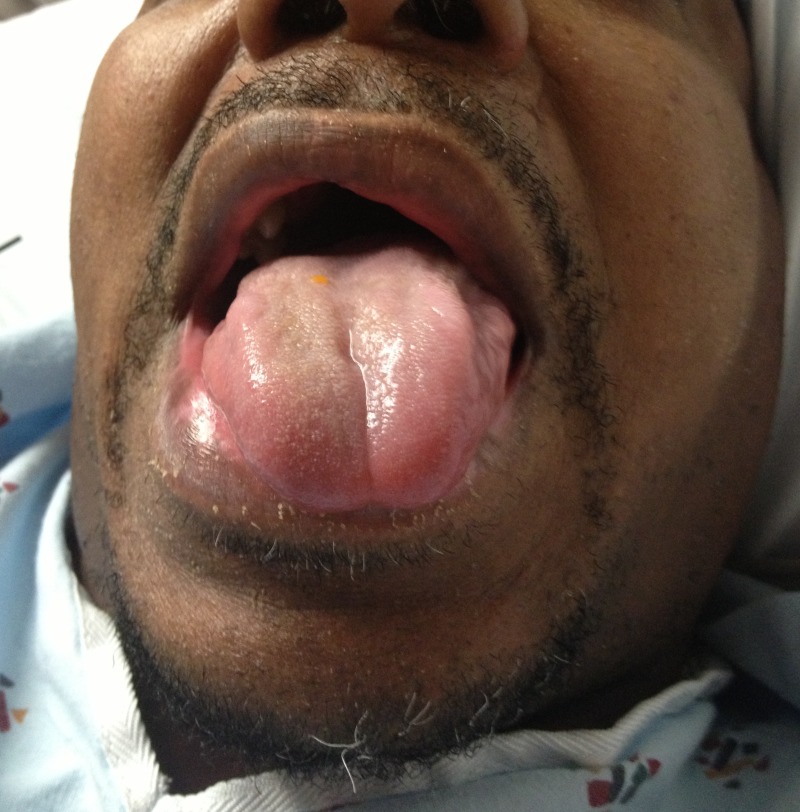
Patient’s tongue appearance after treatment.

Per protocol he was evaluated by a neurologist and received intravenous (IV) tPA 90mg, 10% as bolus and the remainder as an IV drip over the following hour. His weakness began to improve. As the tPA infusion was ending, the patient started complaining of tongue swelling and his voice was noticed to be altered. He did not have any shortness of breath. On examination the patient had a significant amount of left-sided dorsal and ventral tongue swelling, which abruptly ended at the midline (Figure 1). The posterior pharynx, uvula, soft palate, floor of mouth did not have any swelling. Otolaryngology was consulted and performed a bedside flexible laryngoscopy. He did have some mild interarytenoid, arytenoid, and superior aspect of esophageal inlet swelling. The vallecula and epiglottis were within normal range. He was started on 10mg of dexamethasone IV, 20mg of famotidine IV, and 50mg of diphenhydramine IV. He remained non-intubated and was transferred to the neurologic intensive care unit. Magnetic resonance imaging/angiography demonstrated restricted diffusion in genu and posterior limb of right internal capsule with some extension into the right cerebral peduncle, mild intracranial atherosclerosis of posterior cerebral arteries and a hypoplastic right vertebral artery. His swelling and dysphonia resolved completely within 48 hours (Figure 2). Afterwards, his hospital course was uncomplicated and he was discharged after five days total to acute rehabilitation on aspirin and pravastatin. His NIHSS remained five; however, his left-sided strength had improved. At follow up one month later he was improved, subjectively reporting to be at 60% of his baseline. On examination his speech was fluent with no dysarthria or aphasia, no tongue swelling, strength was 5/5 in all four extremities, although he did have mild dysmetria on the left during finger-to-nose.

## DISCUSSION

TPA is a thrombolytic drug used in the treatment of acute strokes. It hydrolyzes plasminogen to plasmin and results in its fibrinolytic effect. The increase in plasmin may play a role in the development of angioedema by activating the kinin pathway and leading to the formation of the vasodilator bradykinin. Plasmin also activates the complement system and leads to the production of the anaphyloxins C3a, C4a, and C5a, which also cause mast cell degranulation and histamine release.^4^ Patient assessment should be done every 15 minutes during tPA infusion for signs of clinical deterioration indicating a possible intracranial hemorrhage, or for signs of angioedema. Angioedema is defined as an acute, transient, well-demarcated swelling that involves the deeper layers of the skin. It usually affects the face, genitalia, as well as the upper respiratory airways and the intestinal epithelial lining.^5^ Angioedema could be due to a hereditary deficiency in C1-esterase or it could occur as an allergic reaction to some medications, most commonly ACE inhibitors. The half-life of tPA is approximately seven minutes; therefore, the risk of angioedema can still occur after the infusion has stopped. Hill et al. conducted a prospective study examining 176 patients treated with tPA for acute ischemic stroke and found evidence of orolingual angioedema in nine patients (5.1%). They reported that the typical reaction was mild, transient, and contralateral to the ischemic hemisphere. The lateralization of the edema was hypothesized to be due to the loss of autonomic innervation of that side.^6^ Engelter et al. in 2005, published a study about the incidence of life-threatening orolingual angioedema during thrombolysis in acute ischemic stroke. They reported two cases (1.7%) of 120 patients treated with alteplase for acute stroke. Of the two cases, one was mild, and impending asphyxia prompted immediate intubation in the second case. In both studies the risk of angioedema was significantly associated with ACE-inhibitor use.^7^ The initial goal of therapy is airway management with early intubation if necessary. Due to the extensive airway swelling that can occur in the setting of angioedema and the possibility for an airway disaster, the most skilled person available must handle airway interventions. The tPA infusion should be stopped. Patients who develop angioedema should be treated with histamine antagonists, such as ranitidine and diphenhydramine along with corticosteroids. Patients are admitted to a neurologic intensive care unit for observation.^8^,^9^ This is an interesting and unique case because our patient was treated with tPA in 2011, without any side effects. It is possible that he formed antibodies to tPA and had a type I allergic reaction two years later when he was being treated for his third stroke. Having been on an ACE inhibitor increases his chances of having angioedema; however, it is likely that repeated exposure to tPA increases the likelihood of angioedema and emergency physicians should be aware of this risk.
